# Determinants of right ventricular systolic dysfunction among patients with left heart failure in a Ghanaian hospital

**DOI:** 10.5830/CVJA-2022-051

**Published:** 2022-09-29

**Authors:** Abdul-Subulr Yakubu, Eugene Amable, Alfred Doku, Francis Agyekum, Alfred Doku, Francis Agyekum

**Affiliations:** Department of Medicine, Tamale Teaching Hospital, Tamale, Ghana; University of Ghana Medical School, Accra, Ghana; Department of Medicine and Therapeutics, Korle Bu Teaching Hospital, Accra, Ghana

**Keywords:** heart failure, right ventricular dysfunction, Ghana

## Abstract

**Background:**

The presence of right ventricular dysfunction affects outcomes in patients with left heart failure. We assessed the determinants of right ventricular systolic dysfunction (RVSD) among patients with left heart failure presenting to the Korle Bu Teaching Hospital of Ghana.

**Methods:**

Consecutive patients with left heart failure who were 18 years and above were prospectively enrolled and assessed for evidence of RVSD by measuring the tricuspid annular plane systolic excursion, the peak velocity of the tricuspid annulus in systole (RV S′), the two-dimensional right ventricular fractional area change (RV FAC) and the right ventricular myocardial performance index (RV MPI).

**Results:**

Two hundred and seventy participants were enrolled, of whom 75.2% had at least one abnormal index of right ventricular systolic function. The prevalence of RVSD was significantly higher among those with non-hypertensive heart failure (85.3 vs 66.0%, p < 0.001). The left ventricular outflow tract velocity–time integral (LVOT VTI) was strongly correlated with the RV FAC and an LVOT VTI < 9.8 cm predicted the presence of an RV FAC < 35% with a sensitivity of 81.5% and specificity of 81.9% [area under the curve 0.882; 95% confidence interval (CI): 0.838–0.926, p < 0.001]. Independent predictors of the presence of RVSD included a transmitral E/A > 2 [odds ratio (OR) = 4.684, 95% CI: 1.521–14.428, p = 0.007), left ventricular ejection fraction < 40% (OR = 4.205, 95% CI: 1.643–10.760, p = 0.003), pulmonary artery systolic pressure (PASP) ≥ 35 mmHg (OR = 2.434, 95% CI: 1.012– 5.852, p = 0.047) and systemic systolic blood pressure (SBP) < 140 mmHg (OR = 2.631, 95% CI: 1.152–6.011, p = 0.022).

**Conclusion:**

RVSD was common in these Ghanaian patients with left heart failure. Left ventricular function, SBP and PASP were independent predictors of the presence of RVSD. Pending further validation, the LVOT VTI may serve as a useful surrogate or screening tool for RVSD in these patients.

Heart failure is a major public health problem in sub-Saharan Africa, where uncontrolled systemic hypertension remains the major cause.^1^,^2^ Heart failure patients with right ventricular (RV) dysfunction report poorer exercise tolerance and have a worse prognosis than patients with preserved RV function.^3^

The aetiology of RV dysfunction in left heart disease is varied. The function of the two ventricles are interdependent and left ventricular (LV) activity contributes substantially to the flow and pressure generated by the right ventricle during systole.^4^ The right ventricle may be involved as part of a global disease involving both ventricles or may suffer as a consequence of increased RV afterload in the context of pulmonary hypertension due to left heart failure.^5^

Historically, little attention has been paid to the study and interpretation of RV function.^6^ Diagnostic or therapeutic recommendations based on RV assessment are scant, mainly due to limited data on the determinants of RV function, mechanisms leading to its failure and its relationship to outcomes. The complex shape of the right ventricle presents inherent challenges in accurately assessing RV dimensions and functional parameters. Echocardiography is a widely available, inexpensive and validated tool for evaluation of the right ventricle.^6^

Risk-stratifying patients with heart failure, based on their RV function, may help identify those patients who are at a particularly high risk of adverse outcomes and help tailor their management. We assessed the clinical and echocardiographic determinants of RV systolic dysfunction (RVSD) in patients with left heart failure with a broad range of LV systolic function.

## Methods

We conducted a cross-sectional study on consecutive patients with left heart failure (HF) presenting for echocardiography at the cardiology unit of the Department of Medicine and Therapeutics of Korle Bu Teaching Hospital (KBTH), Ghana, from January 2020 to June 2021. Inclusion criteria were age ≥ 18 years and a clinical diagnosis of left heart failure who fulfilled the Framingham criteria for the diagnosis of HF and who had satisfactory images on transthoracic echocardiography (TTE).^7^

Patients were excluded if there was another competing reason for their symptoms other than left HF or had no LV systolic or diastolic dysfunction or relevant structural left heart disease at echocardiography. Also excluded were patients with causes of elevated pulmonary pressures other than left heart disease, including those with known chronic interstitial lung disease, chronic obstructive pulmonary disease, bronchial asthma, primary pulmonary arterial hypertension and congenital heart disease. Applying a prevalence of RVSD of 19.7% among HF patients and a 5% margin of error, a minimum sample size requirement of 244 patients was estimated.^8^

Clinical and demographic data, including co-morbidities, medication use and clinical features of HF were collected. Each participant had a 12-lead electrocardiogram (ECG) performed and interpreted using standard criteria.^9^ All participants had standard two-dimensional (2D), M-mode, and Doppler TTE studies performed using a commercially available ultrasound machine (Vivid T8 dimension ultrasound imaging system, GE Healthcare Bio-sciences Corp, Piscataway, NJ, USA) equipped with a 3.5-MHz phased array probe with the patient in the left lateral decubitus position. Measurements were taken in accordance with the recommendations of the American Society of Echocardiography.^10^

Each echocardiogram was analysed for pericardial disease, chamber size and wall dimension, wall motion abnormalities, ventricular function, valve morphology and function, intramural thrombi and congenital defects. For patients in atrial fibrillation, all measurements were averaged over five cardiac cycles, based on the guideline.^11^

RV systolic function was assessed using the tricuspid annular plane systolic excursion (TAPSE), the peak velocity of the tricuspid annulus in systole (RV S′), the 2D right ventricular fractional area change (RV FAC) and the right ventricular myocardial performance index (RV MPI).12 The presence of RVSD was suggested by the detection of at least one abnormal measurement of these parameters.

TAPSE was measured in the standard apical four-chamber view by placing the M-mode cursor through the lateral tricuspid annulus under 2D echocardiographic guidance and measuring the maximum systolic excursion of the lateral tricuspid annulus during held end-expiration. A TAPSE value of < 1.6 cm was considered evidence of impaired RV systolic function. The RV S′ was obtained using pulsed tissue Doppler by placing the sample volume on the lateral tricuspid annulus in the apical four-chamber view during held end-expiration with minimal angulation. The highest systolic velocity (RV S′) was recorded and values < 10 cm/s represented evidence of impaired RV systolic function.

From the same pulsed tissue Doppler signal, the tricuspid closure–opening time (TCOT) and the tricuspid valve ejection time (TV ET) were measured and the RV MPI was calculated as the ratio:

RV MPI = (TCOT–TV ET)/TV ET.

An RV MPI of > 0.55 represented evidence of RV dysfunction.^12^

The RV FAC was obtained by tracing the RV endocardium in the apical four-chamber view with a focus on the right ventricle at end-diastole and end-systole. Two-dimensional RV FAC was calculated as follows:

RV FAC = [(RV end-diastolic area – RV end-systolic area)/ end-diastolic area] × 100.

A value < 35% was taken as evidence of RV systolic dysfunction.^12^

Participants with a left ventricular ejection fraction (LVEF) of at least 50% and evidence of LV diastolic dysfunction were classified as HF with preserved ejection fraction (HFpEF). Those with a LVEF < 40% were classified as HF with reduced ejection fraction (HFrEF), while those with a LVEF in the range of 40–49% were classified as HF with mildly reduced ejection fraction (HFmrEF). The aetiology of the left HF was decided on the basis of the history, physical examination, ECG and echocardiography, as well as a review of relevant investigations, according to the guidelines of the European Society of Cardiology on the diagnosis and treatment of HF.^13^

The study complied with the principles outlined in the Declaration of Helsinki on the ethical principles for medical research involving human subjects. The study protocol was approved by the KBTH Institutional Review Board (protocol number KBTH-IRB/000121/2019).

## Statistical analysis

Categorical variables, presented as counts and percentages, were compared using the chi-squared (χ2) or Fisher’s exact test, as appropriate. Continuous variables are summarised as their means with standard deviation or as medians with interquartile ranges. The means of two groups of continuous variables were compared with the independent samples Student’s t-test for normally distributed data or the Mann–Whitney U-test for skewed data. The associations between continuous variables were assessed by the Pearson’s correlation coefficient.

Multivariate regression models were constructed to assess the relationship of RVSD and other variables that showed a statistically significant correlation with RV FAC, TAPSE, RV S′ and RV MPI in the univariate analyses at an alpha level of 0.05. The variables tested include those hypothesised to contribute to RV dysfunction, including heart failure aetiology, heart rhythm, LV function, pulmonary pressure and HF therapy. Receiver operating characteristic (ROC) curve analysis was employed as appropriate to evaluate the discriminatory value of echocardiographic variables that showed a significant independent predictive value for RVSD. The optimal cut-off value was chosen as the value maximising sensitivity plus specificity.

All tests were two-tailed and a p-value < 0.05 was regarded as statistically significant. Data transformations and analysis were performed with the Statistical Package for the Social Sciences version 20.0 software (SPSS, IBM Corporation, Armonk, NY, USA).

## Results

A total of 270 participants were recruited for the study, of whom 132 (48.9%) were female. Among those with HFrEF, 53.3% were taking a renin–angiotensin system inhibitor, 35.6% were on beta-blockers and 28.1% took mineralocorticoid receptor antagonists. The median duration of treatment received before enrolment was 2.0 weeks (0.0–24.0).Table 1 compares the baseline characteristic of the participants based on the presence or absence of RVSD.

**Table 1:** Clinical characteristics of participants according to RV systolic function

*Variable*	*Total (n=270) (100%)*	*No RVSD (n = 67) (24.8%)*	*RVSD (n=203) (75.2%)*	*p-value*
Age, years	57.0 + 15.7	59.5 + 14.5	56.2 + 16.0	0.126
Gender (female), n (%)	2 (48.9)	33 (49.3)	99 (48.8)	0.945
Diabetes mellitus, n (%)	59 (21.9)	14 (20.9)	45 (22.2)	0.827
Hypertension, n (%)	191 (70.7)	56 (83.6)	135 (66.5)	0.008*
Dyslipidaemia, n (%)	60 (22.2)	2(17.9)	(23.6)	0.328
Ever smoker, n (%)	29 (10.7)	9 (13.4)	20 (9.9)	0.412
Alcohol use, n (%)	84 (31.1)	24 (35.8)	60 (29.6)	0.337
NYHA class, n (%)				< 0.001*
I	10 (3.7)	5 (7.5)	5 (2.5)	
II	76 (28.1)	(52.2)	41 (20.2)	
III	83 (30.7)	17 (25.4)	66 (32.5)	
IV	101 (37.4%)	10 (14.9%)	91 (44.8)	
SBP (mmHg)	131.0 + 30.4	149 + 36	125 2 66	< 0.001*
DBP (mmHg)	83.0 + 20.5	89 + 24	81 + 19	0.004*
Weight, kg	72.0 (66.0-83.0)73.0	(68.0-80.0)71.0	(66.0-83.0)	0.739
Height, cm	168.0 (165.0-170.0)	168.0 (165.0-170.0)	168.0 (165.0-170.0)	0.556
BMI, kg/m²	26.39 + 4.41	26.8 + 5.0	26.3 + 4.2	0.361
BSA, m²	1.85 + 0.17	1.87 + 0.17	1.85 + 0.17	0.447
Treatment, n (%)				
ACEI	73 (27.0)	(31.3)	(25.6)	0.360
ARB	(15.9)	9 (13.4)	(16.7)	0.520
ARNI	11 (4.1)	1 (1.5)	10 (4.9)	0.302
Beta-blocker	96 (35.6)	4(35.8)	72 (35.5)	0.958
MRA	(19.6)	4 (6.0)	49 (24.1)	0.001*
Loop diuretics	184 (68.1)	38 (56.7)	146 (71.9)	0.021*

ACEI, angiotensin converting enzyme inhibitor; ARB, angiotensin receptor

blocker; ARNI, angiotensin receptor–neprilysin inhibitor; BMI, body mass

index; BSA, body surface area; DBP, diastolic blood pressure; MRA, mineralocorticoid

receptor antagonist; NYHA, New York Heart Association functional

class; SBP, systolic blood pressure.

All values are presented as mean ± SD, *n *(%) or median (interquartile range).

*p-value is statistically significant.

The rhythm was sinus in 90.7% of the participants and 7.0% had atrial fibrillation or flutter. Repolarisation abnormality, comprising abnormal ST-segment deviation and/or T-wave abnormalities, was a common finding (93.0%) and was present in a significantly higher proportion of those with RVSD (p < 0.001). Overall, 94.0% of those without RVSD and 99.0% of those with RVSD were judged to have abnormal ECGs (p = 0.035).

HFrEF was present in 146 of the participants (54.1%) while 31 (11.5%) and 93 (34.4%) had HFmrEF and HFpEF, respectively. Table 2 summarises the echocardiographic characteristics based on the presence or absence of RVSD. LVEF was significantly lower in those with RVSD. Measures of diastolic function (mitral and tricuspid E/A and E/e′ ratios and deceleration time) were significantly worse in those with RVSD. Pulmonary hypertension, defined as a PASP > 35 mmHg, was present in 84.6% of those with RVSD and 48.9% of those without RVSD (p < 0.001).

**Table 2:** Echocardiographic characteristics of the participants with and without RVSD

	*No RVSD*	*RVSD*	
*Variable*	*(n=67) (24.8%)*	*(n=203) (75.2%)*	*p-value*
Left atrial area, cm²	24.5 + 6.2	26.0 + 6.7	0.106
Right atrial area, cm2	17.7 + 6.1	22.8 + 8.5	0.001*
Left atrial volume index, ml/m²	45.4 + 17.5	53.4 + 25.7	0.020*
LV end-diastolic diameter, cm	5.3 + 1.0	5.8 + 1.2	0.001*
LV end-systolic diameter, cm	3.8 + 1.1	4.7 + 1.4	< 0.001*
LV mass index, g/m²	138.0 + 49.6	137.9 + 51.5	0.997
Relative wall thickness	0.48 + 0.17	0.41 + 0.22	0.007*
LV end-diastolic volume, ml	119.4 + 57.7	157.7 + 78.5	0.001*
LV end-systolic volume, ml	62.9 + 48.7	107.9 + 69.3	<0.001*
LV ejection fraction (%)	52.7 + 16.3	36.0 + 18.2	<0.001*
LVOT VTI, cm	16.7 + 6.4	10.8 + 5.7	<0.001*
RV basal diameter, cm	4.0 + 0.8	4.5 + 1.0	0.002*
RV mid-diameter, cm	+ 0.5	3.2 +0.9	0.001*
RV longitudinal diameter, cm	6.8 + 1.2	7.3 3 1.4	0.011*
RVOT diameter, cm	2.8 + 0.5	3.2 + 0.7	0.001*
RV end-diastolic area, cm²	17.7 + 6.2	21.7 + 8.6	0.002*
RV end-systolic area, cm²	9.0 + 3.7	14.6 + 7.4	<0.001*
MV E/A ratio	1.2 + 0.8	2.2 + 1.1	<0.001*
MV E/e' ratio	14.1 + 6.8	18.6 + 7.7	<0.001*
MV DT, ms	187 + 73	126 + 53	<0.001*
TV E/A ratio	1.0 + 0.7	1.7 0.9	<0.001*
TV E/e' ratio	7.5 + 4.6	10.6 + 6.2	0.015*
TV DT, ms	207 + 66	152 + 56	<0.001*
PASP, mmHg	37.5 + 14.3	49.0 + 15.9	<0.001*

DT, deceleration time; E/A, early to late diastolic ventricular filling velocity

ratio; E/e′, early diastolic filling velocity to early diastolic annular velocity ratio;

RV FAC, right ventricular fractional area change; LVOT VTI, left ventricular

outflow tract velocity–time integral; RV MPI, right ventricular myocardial

performance index; MV, mitral valve; PASP, pulmonary artery systolic pressure;

RVOT, right ventricular outflow tract; RVSD, right ventricular systolic dysfunction;

TAPSE, tricuspid annular plane systolic excursion; TV, tricuspid valve.

All values are presented as mean ± SD. *p-value is statistically significant.

**Fig. 1:**
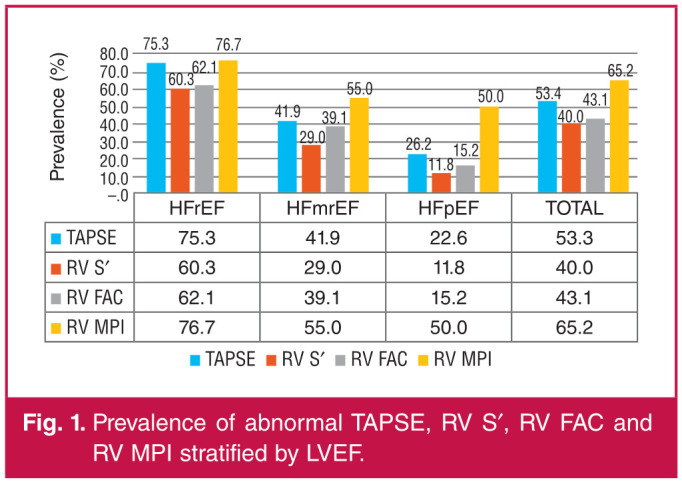
Prevalence of abnormal TAPSE, RV S′, RV FAC and RV MPI stratified by LVEF.

Overall, 203 (75.2%) of the participants had at least one abnormal index of RV systolic function. Abnormal TAPSE was present in 53.3% of participants while abnormal RV S′ and RV FAC were present in 40.0 and 43.1% of participants, respectively (Fig. 1). RV global function, as assessed by the RV MPI, was abnormal (> 0.55) in 65.2% of the participants. Seventy-six (28.1%) had abnormal values for all the indices of RVSD. All the measures of RV systolic function were significantly worse at lower LVEF.

Table 3 summarises the likely HF aetiology of the study participants. Hypertensive heart disease was the predominant cause of HF, responsible for 52.2% of cases. Among participants with dilated cardiomyopathy (DCM), no obvious cause was found in 83.0% of the cases, while 7.5% were alcohol related. Peripartum cardiomyopathy was diagnosed in 5.9% of the study participants. Rheumatic mitral or aortic valve disease was the commonest primary valvular cause of HF, accounting for 62.5% of the cases of primary valvular heart disease causing left HF.

**Table 3:** Aetiology of heart failure among the study participants

*Characteristics*	*Count*	*Percentage*
Aetiology of heart failure		
Hypertensive heart disease	141	52.2
Dilated cardiomyopahy	53	19.6
Ischaemic heart disease	26	9.6
Peripartum cardiomyopathy	16	5.9
Valvular heart disease	16	5.9
Infiltrative and other restrictive cardiomyopathies	8	3.0
Others	10	3.7
Total	270	100.0
Type of dilated cardiomyopathy		
Idiopathic	44	83.0
Alcohol-related	4	7.5
Chemotherapy-associated	2	3.8
Suspected myocarditis	2	3.8
Thyroid disease-associated	1	1.9
Total	53	100.0
Type of valvular heart disease Rheumatic	10	62.5
Endocarditis	3	18.8
Calcific/degenerative	2	12.5
Mitral valve prolapse	1	6.3
Total	16	100.0

aIncluded three cases of cancer therapeutics-related cardiac dysfunction in breast

cancer patients taking anthracyclines, three cases of suspected myocarditis, one

case of hypertrophic cardiomyopathy and three cases of left ventricular systolic/

diastolic dysfunction of uncertain aetiology.

The prevalence of RVSD in those with non-hypertensive HF was significantly higher than in those with hypertensive HF (85.3 vs 66.0%, p < 0.001). The RV MPI was worse in those with an ischaemic aetiology of their HF (p = 0.041). However, the values of TAPSE, RV S′ and RV FAC did not differ based on ischaemic versus non-ischaemic aetiology of the HF.

The Pearson’s correlation coefficients between various echocardiographic measures of LV systolic and diastolic function and the RV FAC, TAPSE, RV S′ and RV MPI are shown in Table 4. The indices of LV systolic function [LVEF and left ventricular outflow tract velocity–time integral (LVOT VTI)] were the strongest correlates of the various measures of RVSD. The LVEF and LVOT VTI showed significant positive correlations with RV FAC, TAPSE and RV S′ and negative correlations with RV MPI.

**Table 4:** Correlations between the indices of RVSD and those of LV systolic and diastolic dysfunction

	*RV*	*FAC*	*TAPSE*		*RV*	*S'*	*RV*	*MPI*
*Variable*	*r*	*p-value*	*r*	*p-value*	*r*	*p-value*	*r*	*p-value*
LVEDV	-0.299	<0.001*	-0.123	0.044*	-0.195	0.002*	0.157	0.022*
LVESV	-0.392	<0.001*	-0.267	<0.001*	-0.330	<0.001*	0.190	0.006*
LVEF	0.540	<0.001*	0.446	< 0.001*	0.561	0.001*	-0.245	0.001*
LVOT VTI	0.657	<0.001*	0.573	< 0.001*	0.542	0.001*	-0.250	<0.001*
MV E/A	-0.437	<0.001*	-0.515	<0.001*	-0.456	<0.001*	0.272	< 0.001*
MV E/e'	-0.314	<0.001*	-0.391	<0.001*	-0.348	<0.001*	0.127	0.093
MV DT	0.413	< 0.001*	0.438	< 0.001*	0.307	< 0.001*	-0.217	0.005*

DT, deceleration time; E/e′, early filling velocity to early diastolic annular velocity ratio; LVEDV, left ventricular end-diastolic volume; LVEF, left ventricular ejection

fraction; LVESV, left ventricular end-systolic volume; LVOT VTI, left ventricular outflow tract velocity–time integral; MV, mitral valve; TV, tricuspid valve.

r = Parson’s correlation coefficient, *p-value is statistically significant.

Multivariate linear regression analyses were performed using variables that showed a significant correlation with RV FAC in the univariate analysis. The final linear regression prediction model for RV FAC contained the LVOT VTI (with log^10^ transformation), RV size as measured at the proximal right ventricular outflow tract (RVOT), the left atrial volume index (LAVI) and the LVEF. The model was statistically significant [F (4, 206) = 79.315, p < 0 .001] and accounted for approximately 59.9% of the variance of RV FAC (R2 = 0.606, adjusted R2 = 0.599). RV FAC was primarily predicted by log^10^ LVOT VTI (β = 40.295, p < 0.001) and to a lesser extent by proximal RVOT diameter (β = –2.858, p = 0.009) and LAVI (β = –0.099, p < 0.001).

**Fig. 2:**
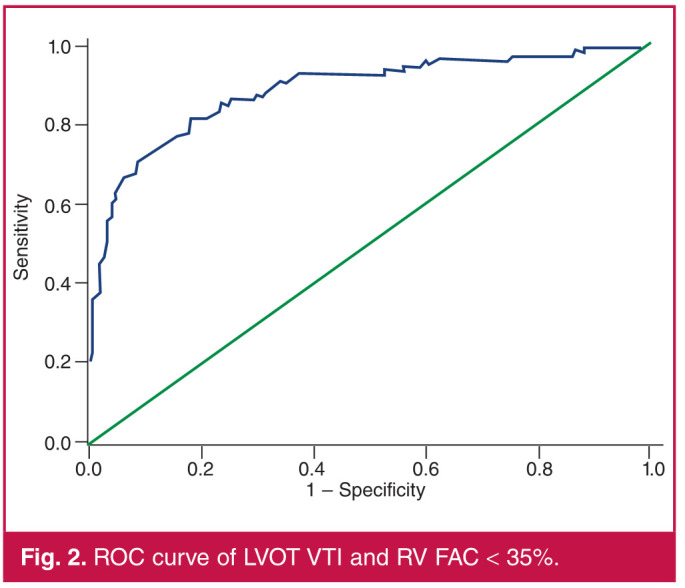
ROC curve of LVOT VTI and RV FAC < 35%.

ROC curve analysis was used to determine the threshold value of LVOT VTI that was associated with RVSD. The ROC analysis defined an LVOT VTI value of < 9.8 cm as the cut-off value for an RV FAC < 35% [area under the curve (AUC) = 0.882; 95% confidence interval (CI): 0.838–0.926, p < 0.001) with a sensitivity of 81.5% and specificity of 81.9% (Fig. 2). Table 5 shows the LVOT VTI cut-off values for the various measures of RVSD.

**Table 5:** Cut-off values for LVOT VTI for the various measures of RVSD on ROC analysis

	*AUC (95% CI)*	*Cut-off value (cm)*	*Sensitivity (%)*	*Specificity (%)*	p-value
RV FAC < 35%	0.882 (0.838-0.926)	9.8	81.5	81.9	<0.001*
TAPSE < 1.6 cm	0.834 (0.787-0.882)	11.4	74.5	71.2	0.001*
RV S' < 10 cm/s	0.832 (0.783-0.880)	10.3	77.1	70.2	< 0.001*
RV MPI > 0.55	0.629 (0.547-0.708)	13.9	69.1	55.6	0.002*
Total RVSD	0.779 (0.721-0.836)	13.6	73.9	68.7	< 0.001*

AUC, area under the curve; CI, confidence interval; RV FAC, right ventricular

fractional area change; TAPSE, tricuspid annular plane systolic excursion;

RV S′, peak velocity of the tricuspid annulus in systole; RV MPI, right ventricular

myocardial performance index; RVSD, right ventricular systolic dysfunction.

*p-value is statistically significant.

Binary logistic regression analysis was performed to ascertain the effects of various clinical and echocardiographic characteristics on the likelihood that the participants would have RVSD, defined as RV FAC < 35%, TAPSE < 1.6 cm, RV S′ < 10 cm/s or RV MPI > 0.55. Only variables that were significant at an alpha level of 0.05 in the univariate analyses were included in the multivariable analysis. The variables tested included age, gender, systolic blood pressure (SBP), New York Heart Association (NYHA) class, body mass index, heart rhythm, aetiology of HF, diastolic parameters (including the transmitral E/A ratio), PASP, LVEF, LAVI and RV size. The final multivariate logistic regression model included LVEF < 40%, mitral valve E/A ratio > 2, PASP ≥ 35 mmHg and SBP < 140 mmHg and was statistically significant [χ2 (4) = 62.921, p < 0.001] (Table 6). The model explained 42.4% of the variance in RVSD and correctly classified 84.5% of cases.

**Table 6:** Independent predictors of RVSD

	*Univariate analysis*		*Multivariate*	*analysis*
*Predictors*	*OR (95% CI)*	*p-value*	*Adjusted OR (95% CI)*	*p-value*
MV E/A > 2	15.172 (6.200-37.127)	< 0.001* 4.684	(1.521-14.428)	0.007*
LVEF < 40%	10.107 (4.974-20.538)	< 0.001* 4.205	(1.643-10.760)	0.003*
PASP 35 mmHg	5.720 (2.830-11.562)	< 0.001* 2.434	(1.012-5.852)	0.047*
SBP < 140 mmHg	4.378 (2.445-7.836)	< 0.001* 2.631	(1.152-6.011)	0.022*
NYHA III/IV	5.056 (2.807-9.108)	< 0.001*	-	-
Male gender	1.020 (0.587-1.771)	0.945	-	-
Obesity	1.288 (0.620-2.678)	0.497	-	-
HR > 90 bpm	2.996 (1.694-5.299)	< 0.001*	-	-
Non-sinus rhythm	1.817 (0.601-5.498)	0.290	-	-
IHD	1.431 (0.517-3.956)	0.490	-	-
HHD	0.335 (0.184-0.609)	< 0.001*	-	-

CI, confidence interval; HHD, hypertensive heart disease; HR, heart rate; IHD,

ischaemic heart disease; LVEF, left ventricular ejection fraction; MV E/A, transmitral

early to late ventricular filling velocity ratio; NYHA, New York Heart

Association; OR, odds ratio; PASP, pulmonary artery systolic pressure; SBP,

systolic blood pressure.

aNagelkerke R2 = 0.424, p < 0.001. *p-value is statistically significant.

## Discussion

The reported prevalence of RVSD in left HF varies with the stage and aetiology of the HF, as well as the definition of RVSD employed.^14^,^15^ The use of several indices of RV function, each measuring a different component of RV contractility, increases the chances of finding RVSD. The prevalence of RVSD was higher when assessed by the RV MPI than by TAPSE, RV S′ or RV FAC for all LVEF subgroups and all HF aetiologies. The RV MPI is said to be an index of global RV function that takes into account both systolic and diastolic RV function and appears to be independent of preload, afterload and heart rate.^16^

There was a significant association between the presence of RVSD and the degree of LV systolic and diastolic dysfunction. Experimental studies have suggested that approximately 20 to 40% of RV systolic function is contributed to by LV contraction through ventricular interdependence.^4^ In agreement with this, LVEF was independently associated with the presence of RVSD in the present study. Overall, an LVEF of < 40% was associated with a four-fold odds ratio (OR) of having RVSD (OR = 4.205, 95% CI: 1.643–10.760, p = 0.003). Similarly, RV systolic function worsened with worsening grade of LV diastolic function, with the prevalence of RVSD reaching 93.2% in those with a restrictive LV filling pattern. A mitral E/A > 2 was independently predictive of RVSD in the multivariable logistic regression analysis (OR = 4.684, 95% CI: 1.521–14.428, p = 0.007).

RV systolic dysfunction was significantly more common in patients with non-hypertensive HF in the present study. Based on cut-off values generated from normal controls, an RVSD prevalence of as high as 81.6% was reported among patients with hypertensive HF in Ibadan, Nigeria.^17^ More normative studies are needed to define the limits of normal RV function in indigenous Africans.

Ischaemic aetiology has been reported to be independently associated with a reduced TAPSE in patients with heart failure.^18^ Kjaergaard et al., in a study among Danish patients, found that patients with ischaemic cardiomyopathy had lower values of TAPSE compared to non-ischaemic aetiology (17 ± 5 vs 19 ± 5 mm, p = 0.001).19 However there is a suggestion that myocardial infarction involving the anterior wall of the left ventricle may produce an increased TAPSE as a compensatory response, while infarctions involving the right ventricle will produce a reduced TAPSE.^20^ In the present study, RV MPI was significantly higher (worse global RV function) in those with ischaemic heart disease. However, HF aetiology was not independently predictive of RVSD in the multivariable analysis.

The presence of an elevated pulmonary pressure leading to afterload mismatch and contractile impairment has traditionally been considered a plausible cause for RV dysfunction in HF patients.^21^ The present study found that a PASP > 35 mmHg was associated with an increased odds of having of RVSD. This finding is consistent with our understanding of RV pulmonary artery coupling and the fact that RV function is highly afterload dependent.^22^ Consistent with that reported by Ghio et al. in a European population, the present study found that the independent association of a PASP > 35 mmHg with RVSD was significant, mainly in those with HFmrEF and HFpEF.^18^

The observation that RVSD was associated with the presence of an elevated RV afterload in HFpEF and HFmrEF is particularly important and provides a strong rationale for discovering effective treatments for pulmonary hypertension in such patients.^23^ However this association between RVSD and pulmonary pressure has not been consistently observed in other studies. The reason for this disparity is not immediately apparent but may be related to the varied aetiology of HF in the participants of the different studies, the definition of pulmonary hypertension and how it was measured.

In our study, SBP had a significant correlation with all measures of RVSD and was independently predictive of RV FAC < 35% and RV S′ < 10 cm/s, mainly for those with HFrEF. The development of RVSD may result in the inability of the cardiovascular system to maintain an adequate blood pressure, even in those who were previously hypertensive. It is well known that in advanced heart failure, SBP is usually low, even in previously hypertensive patients.^24^

This phenomenon, which has been termed decapitated hypertension, occurs when patients who were previously hypertensive progressively develop normal and even low blood pressure as HF worsens.^25^ This decrease in SBP results from reduced pump function and a fall in cardiac output despite compensatory peripheral vasoconstriction.^24^ While hypertension is an established cause of incident HF, studies have shown that a higher SBP in patients with established heart failure seems to paradoxically have a protective effect on survival.^26^

On multivariate linear analysis, LVOT VTI, LAVI, RVOT diameter and LVEF demonstrated a significant relationship with the measured RV FAC. The LVOT VTI was particularly well correlated with the RV FAC and was the strongest independent predictor of RV FAC in the final multivariate linear regression model. The LVOT VTI, a Doppler-derived measure of the distance travelled by midstream blood through the LV outflow tract in a single cardiac cycle, is a relatively simple parameter that serves as a proxy estimate of forward stroke volume.^27^ It is easily reproducible with low interobserver variability and can be incorporated into a routine echocardiographic examination with little additional resources or time.^28^ In our study, an LVOT VTI < 9.8 cm in patients with left HF was found on ROC analysis to be the cut-off value for a RV FAC < 35% with a sensitivity of 81.5% and specificity of 81.9%.

Apart from RV FAC, RVSD measured by RV S′, TAPSE and RV MPI were significantly worse in males with left HF compared with females. Other studies have previously reported no gender difference in RVSD.^29^ The reason for the gender disparity in RV S′, TAPSE and RV MPI observed in the current study is not immediately apparent and is not explained by HF severity or aetiology. A possible reason is the varied cut-off values used to define RVSD in these studies. This underscores the importance of more normative studies to define the optimal cut-off limits of normal RV function in indigenous Africans. We did not find an association between patient’s age and the presence of RVSD in this study.

The study had some limitations. Being hospital-based and involving a single centre limits the generalisability of the results. Cardiac magnetic resonance imaging (MRI) has emerged as the most accurate technique to quantify RV size and function.^30^ However, the cost implications and limited access to cardiac MRI make it less suitable for routine clinical purposes in a resourceconstrained setting. Echocardiography, despite its limitations, is widely available, affordable and has acceptable sensitivity.

Determination of HF aetiology was based on clinical features, ECG and echocardiographic findings. Information about coronary anatomy and functional ischaemia tests were not routinely available so it is conceivable that some participants with significant ischaemic heart disease were not identified as such. Additionally, TAPSE is not a validated index for assessing RV function in the presence of atrial fibrillation. While the number of patients with atrial fibrillation in this study was relatively low (7.0%), this limitation must be borne in mind in interpreting findings that are based on TAPSE measurements.

## Conclusion

RV systolic dysfunction was common in these Ghanaian patients with left HF. Worsening LV systolic and diastolic function, higher PASP and a low systemic SBP were predictors of the presence of RVSD, which was more common among patients with non-hypertensive HF. Further studies may help determine the optimal cut-off value for LVOT VTI that would reliably predict the presence of RVSD in patients with left HF. 
